# Two Dyscalculia Subtypes With Similar, Low Comorbidity Profiles: A Mixture Model Analysis

**DOI:** 10.3389/fpsyg.2021.589506

**Published:** 2021-06-21

**Authors:** Christian Kißler, Christin Schwenk, Jörg-Tobias Kuhn

**Affiliations:** Methods of Educational Research, Faculty of Rehabilitation Sciences, TU Dortmund University, Dortmund, Germany

**Keywords:** subtypes, mathematical skills, mathematical abilities, mixture model analysis, comorbidity, dyscalculia, developmental dyscalculia

## Abstract

Several studies have aimed to identify subtypes of dyscalculia. In many of these studies, either pre-defined groups (e.g., children with reading and mathematical difficulties vs. children with isolated mathematical difficulties) were analyzed regarding their cognitive profiles (top-down approach), or clusters of children with dyscalculia (CwD) were identified based on a narrow range of cognitive and mathematical skills (data-driven or bottom-up approach). However, it has remained difficult to establish robust subtypes of dyscalculia across studies. Against this background, we conducted a mixture model analysis in order to explore and identify subtypes of dyscalculia based on a broad range of variables (intelligence, reading fluency, working memory, attention, and various mathematical skills). The total sample comprised 174 elementary school CwD (IQ > 70; mathematical abilities: percentile rank <10), which consisted of two subsamples. The first subsample was based on a diagnostic test focusing on calculation (HRT 1–4; *n* = 71; 46 girls, 25 boys; age: *M* = 9.28 years, *SD* = 0.94) whereas the second subsample was based on a diagnostic test with a strong focus on basic numerical capacities (ZAREKI-R; *n* = 103; 78 girls, 25 boys; age: *M* = 8.94 years, *SD* = 1.05). Results provided convincing evidence for the existence of two subtypes in CwD: A slightly impaired subtype and a strongly impaired subtype. Subtypes differed most strongly regarding mathematical abilities, but the analyses suggest that differences in attention could also be a key factor. Therefore, comorbid attention difficulties seem to be a relevant factor that needs to be considered when establishing subtypes. Substantial intelligence differences between dyscalculia subtypes could not be found. Differences in working memory and reading fluency were negligible. Overall, the results seemed to be robust regardless of the diagnostic test used for assessing dyscalculia. When planning interventions for CwD, the existence of a subtype with substantial attention problems should be kept in mind.

## Introduction

Mathematical skills are important for a successful biography: For example, there is a strong connection between mathematical skills in childhood and adult socioeconomic status (Ritchie and Bates, 2013). Therefore, children with difficulties in mathematics face the risk of serious consequences. Dyscalculia is defined as an impairment of basic arithmetic skills (addition, subtraction, multiplication, division), which cannot solely be explained by a general intelligence deficit nor by inadequate learning environment (ICD-10: Dilling et al., 1993). In the ICD-11, (developmental) dyscalculia is described as a developmental learning disorder that is characterized by a lack of “skills related to mathematics or arithmetic, such as number sense, memorization of number facts, accurate calculation, fluent calculation, and accurate mathematic reasoning” (World Health Organization, 2020). In any case, mathematical skills and mastery of mathematical procedures, along with mathematical fact retrieval, are strongly impaired in children with dyscalculia (CwD) (Geary et al., 2012; Kuhn et al., 2013; Mazzocco et al., 2013). Landerl et al. (2004, p. 99) “conclude that dyscalculia is the result of specific disabilities in basic numerical processing rather than the consequence of deficits in other cognitive abilities.” *Basic numerical processing* (BNP) has also been referred to as core number competencies and is assessed using simple tasks such as dot enumeration and comparison of single digits (Reeve et al., 2012). In addition, there are more complex mathematical precursor skills (*complex number processing*, CNP): For example, mental number line tasks, which require participants to locate a given number on a number line, or the ability to convert auditorily presented numbers into written Arabic symbols (transcoding; Nuerk et al., 2006; Kuhn et al., 2013, 2017). Deficits in the processing of numbers and/or magnitudes are discussed as the main causes of dyscalculia (Butterworth, 2005; Noël and Rousselle, 2011; Moll et al., 2015). Overall, different mathematical skills can be impaired in CwD: Individual profiles and therefore problem areas and needs can vary substantially across individuals (Haberstroh and Schulte-Körne, 2019). Therefore, different subtypes of CwD might exist which display different profiles concerning BNP, CNP, and calculation skills.

In fact, arithmetic errors of CwD vary with their cognitive profile (Rourke, 1993, p. 218). Because error patterns, at least partially, reflect strategy use or selective deficits and thus can be relevant starting points for interventions, several studies have aimed to identify subtypes (or subgroups) of CwD (e.g., Geary, 1993; Von Aster, 2000; Bartelet et al., 2014; Skagerlund and Träff, 2016). When trying to identify subgroups of CwD, a broad range of cognitive abilities has to be taken into account because mathematical skills rely on many different cognitive abilities.

Although some etiological views (e.g., Landerl et al., 2004) focus on domain-specific causes of dyscalculia, mathematical deficits in the heterogeneous population of CwD can be based on additional, domain-general causes. For example, the mathematical deficits of some CwD seem to be associated with impairments in verbal short-term memory (Szücs, 2016). Further, it is widely known that a large number of children with mathematical deficits also display impairments in reading or attention (Gross-Tsur et al., 1996; Willburger et al., 2008; Haberstroh and Schulte-Körne, 2019). In addition, many CwD display difficulties in working memory (e.g., Keeler and Swanson, 2001; Schuchardt et al., 2008; Mähler and Schuchardt, 2011). According to Baddeley’s (1992) framework, on which the majority of working memory assessments are based, working memory is divided into three structural parts. One part (the central executive) coordinates the storage and manipulation of the information, whereas two other parts (slave systems) are responsible for storing (1) auditory (phonological loop) or (2) visuo-spatial information (visuo-spatial sketchpad; Baddeley, 1992; Cragg et al., 2017). Many studies report that CwD display deficits in the visuo-spatial sketchpad (e.g., Schuchardt et al., 2008). However, not all studies replicated that CwD have significant difficulties in working memory (e.g., Landerl et al., 2009; Kißler et al., 2021). Specifically, Landerl et al. (2009) did not find significant deficits regarding block tapping tasks (*Corsi block* tapping task: e.g., Berch et al., 1998) when comparing CwD with a control group. However, children with dyscalculia and comorbid reading difficulties performed significantly lower than a control group. Hence, stronger or more diverse working memory difficulties could be linked to comorbidity, i.e., to different subtypes of CwD with or without comorbid impairments. Correlative findings corroborate this assumption (e.g., Peng et al., 2016). In sum, there is some evidence for different subtypes of CwD that are characterized by varying deficits in working memory.

As mentioned earlier, attention problems and reading difficulties are often associated with dyscalculia, but not all CwD seem to have these problems (e.g., Gross-Tsur et al., 1996; Haberstroh and Schulte-Körne, 2019). Correspondingly, attention deficits and dyslexia could also be factors that need to be considered when discussing dyscalculia subtypes. In the past, it was common to differentiate between children who showed a discrepancy between general intelligence and individual calculation or reading performance, and children who did not show such an intelligence discrepancy (e.g., Dilling et al., 1993). Even if the intelligence discrepancy criterion is no longer recommended for diagnosing CwD because of methodological and content-related reasons (e.g., Ehlert et al., 2012; Kuhn et al., 2013), intelligence should still be taken into account as a further factor in a holistic typification of CwD.

After describing cognitive features (attention, intelligence, reading skills, working memory, and different arithmetic abilities and skills as BNP, CNP, and calculation) that are often associated with dyscalculia and that may vary across subtypes of CwD, the next part of this introduction focuses on different methodological approaches and conclusions of studies conducted in this field. In order to identify subtypes of dyscalculia, two different approaches have been pursued: Some studies analyzed predefined subtypes based on specific theoretical expectations, whereas others used a data-driven approach and therefore tended to be more exploratory.

Some of the first studies analyzing predefined subtypes were conducted by Rourke and his research team (e.g., Ozols and Rourke, 1988; Rourke, 1993). These authors divided CwD into three groups: (1) children with problems in arithmetic, reading and spelling, (2) children with deficient reading and spelling abilities who displayed higher (albeit still deficient) arithmetic skills, and (3) children with average or above reading and spelling performance, but with mathematical problems. Arithmetic errors of these groups varied in a qualitative way: For example, while children in group 2 mostly made mistakes that could be related to their reading problems, children in group 3 showed a broad range of mechanical arithmetic errors (Rourke, 1993). Children in group 3 had problems to calculate correctly because of their poor handwriting; they misread the mathematical signs, they performed arithmetic operations incorrectly and they had problems to access the needed calculation rules from long-term memory (Rourke, 1993). Thus, this line of research provided evidence that subtypes of children with problems in arithmetic differ depending on their reading skills. This finding underscores the importance of reading skills when describing subtypes of dyscalculia.

In an early review, Geary (1993) also described three different subtypes of CwD. In contrast to Rourke (1993), Geary (1993) did not focus on the comorbidity of dyscalculia and reading/spelling disorder: One of these subtypes displayed “difficulties in arithmetic fact retrieval and problems in the memorization of arithmetic tables even with extensive drilling” (Geary, 1993, p. 357). Furthermore, he described a second subtype with “difficulties in the use of arithmetical procedures” (p. 357). The third subtype described by Geary (1993) had visuospatial difficulties and consequently, this subtype had problems with the processing of numerical information. The subtypes suggested by Geary (1993) relate to difficulties in memory, visuospatial skills, and procedural calculation, thus focusing more strongly on general cognitive abilities when identifying subtypes of dyscalculia.

In a more recent study, Skagerlund and Träff (2016) divided CwD into two subgroups (based on theoretical assumptions) and analyzed them by focusing on different mathematical abilities. These authors described a subtype (*general dyscalculia subtype*) with problems in the innate approximate number system (ANS). In addition, they postulated and found a second subtype with *arithmetic fact dyscalculia*. The latter subtype showed no difficulties in non-symbolic number processing but had difficulties in symbolic number processing. Skagerlund and Träff (2016) concluded that this second subtype is characterized by suffering from a deficit in accessing information from symbols which has been referred to as *access deficit* in the literature (Rousselle and Noël, 2007). In summary, the results of Geary (1993) and Skagerlund and Träff (2016) suggest the existence of subtypes in CwD that differ in their profiles of numerical and arithmetic skills.

Next, studies that used data-driven methods to identify subtypes of dyscalculia are presented. In contrast to the studies analyzing predefined subtypes of dyscalculia, Von Aster (2000) used a data-driven approach (cluster analysis) for subtyping 93 children with poor achievement in school mathematics. In line with the results reported by Rourke (1993), Von Aster (2000) characterized a *verbal subtype* with language-based problems. Von Aster (2000) differentiated this subtype from two other subtypes specific to his study: An *Arabic subtype* with difficulties in understanding and using the Arabic notation system as well as a *pervasive subtype* with strong problems in most mathematical subareas (e.g., a lack of basic numerosity and number concepts).

Similar to Von Aster (2000), Bartelet et al. (2014) also used a data-driven approach. Bartelet et al. (2014) focused on various variables that represent specific cognitive abilities and skills: Spatial short-term working memory, verbal short-term working memory, intelligence, Arabic numeral knowledge, number line estimation, approximate numerical knowledge (e.g., dot comparison task), and counting. Bartelet et al. (2014) identified and described six subtypes of dyscalculia with different cognitive profiles: (1) the *weak mental number line subtype* with a low performance in number line tasks but a high performance in approximate numerical knowledge and Arabic numeral knowledge, (2) the *weak ANS subtype* with problems in approximate numerical knowledge and number line tasks, but with a strong performance in spatial short-term working memory and with a higher IQ in comparison to other subtypes—the characteristics of this subtype resemble the general dyscalculia subtype described by Skagerlund and Träff (2016), (3) the *spatial difficulties subtype* with particular difficulties in spatial short-term working memory and in approximate numerical knowledge, but also difficulties in verbal short-term working memory and number line tasks, (4) the *access deficit subtype* with problems in counting and Arabic numerical knowledge, (5) the *no numerical cognitive deficit subtype* with no deficits in any area and very high verbal short-term working memory, and (6) the *garden variety subtype* with many smaller deficits in different areas, a high performance in number line tasks and a lower IQ. These results suggest a large (and almost confusing) variety of subtypes. It is also noticeable that the characteristics of some subtypes overlap, and that IQ seems to be an important domain-general factor which helps to characterize different subtypes.

In another data-driven subtyping study, Chan and Wong (2020) used a cluster analysis for subtyping CwD over the first 2 years of elementary school to compare the cognitive profiles of the identified subgroups. These authors assessed a broader range of variables compared to many prior studies (working memory and mathematical abilities): Backward digit span, backward block span, the acuity of the ANS, number comparison, number line estimation, number fact retrieval, accuracy in calculation, strategic counting, arithmetic word problems, and a general learning achievement test in mathematics (based on the curriculum of Hong Kong). Moreover, dot enumeration tasks were used to assess both the ability to subitize (1–3 dots) and to enumerate (4–9 dots). Chan and Wong (2020) described five different subtypes of CwD: (1) the *numerosity coding deficit subtype*, (2) the *symbolic deficit subtype*, (3) the *working memory deficit subtype*, (4) the *number sense deficit subtype*, and (5) the *mild difficulty subtype* with almost no deficits in the cognitive areas examined but with some problems in mathematics. The subtypes presented by Chan and Wong (2020) seem to differ from the subtypes described by Bartelet et al. (2014), with some overlaps. The *mild difficulty group* shares some features with the *garden variety subtype* identified by Bartelet et al. (2014), for example—but they are far from being identical. Both studies have in common that they present at least one subtype that is characterized by substantial deficits in working memory.

A recent data-driven study of Huijsmans et al. (2020) aimed to identify different cognitive profiles of 281 fourth graders by examining basic arithmetic and advanced mathematic skills. In contrast to Bartelet et al. (2014) and Chan and Wong (2020), this study did not exclusively focus on CwD. Huijsmans et al. (2020) found four different profiles. Three of those profiles did not seem to have significant mathematical difficulties (a *high-achieving profile*, an *average profile*, and a *divergent profile*). Another profile seemed to have mathematical difficulties in some way (a *low-achieving profile*), but none of the detected profiles met the criteria for dyscalculia. No subgroups of CwD were found. Huijsmans et al. (2020) concluded that the group of CwD could be “too heterogeneous to distinguish subgroups” (p. 9).

The assumption that there are many and very heterogeneous cognitive profiles in CwD (Huijsmans et al., 2020) is in line with the fact that other studies (Bartelet et al., 2014; Chan and Wong, 2020) found a relatively large number of subtypes in CwD. In summary, Bartelet et al. (2014) and Chan and Wong (2020) assessed children’s mathematical performance and their working memory capacity, but only Bartelet et al. (2014) took intelligence into account. In contrast to Rourke (1993), data-driven approaches often neglected reading deficits when subtyping CwD. Therefore, important information for subtyping CwD may have been overlooked. However, as mentioned before, CwD have difficulties in many cognitive areas. The studies by Bartelet et al. (2014) and Chan and Wong (2020) present different typologies, and these differences could be due to the fact that the cognitive profiles of CwD were not considered exhaustively. To systematically overcome heterogeneous, and therefore inconclusive, evidence, it is necessary to consider a broader range of variables and cognitive areas when following data-driven approaches to subtype CwD. Therefore, the present study includes attention, reading fluency, and intelligence beyond working memory and mathematical skills.

It is important to bear in mind that different tests and assessments are used to assess dyscalculia. This may affect results because different groups of children are identified as dyscalculic, depending on the structure of the test used. There is no “gold standard” test for dyscalculia; instead, instruments and diagnostic thresholds depend on (dynamic) consent. Hence, in order to provide more robust results, two different assessments of dyscalculia were used in this study, with an emphasis on different aspects of mathematical difficulties. The first assessment, ZAREKI-R (von Aster et al., 2006), mainly focuses on basic numerical processing (such as the comparison of quantities) and complex number processing (such as number line estimation, or transcoding). In contrast, HRT 1–4 (Haffner et al., 2005) mainly focuses on calculation and arithmetic (e.g., basic arithmetic operations), i.e., on a higher level of mathematical skills. Both tests include addition and subtraction tasks, but only the HRT 1–4 includes tasks where children have to divide and multiply. While ZAREKI-R tests mathematical precursor abilities such as number line estimation, HRT 1–4 includes tasks on visual/geometrical skills such as lengths estimation. In this study, therefore, two different dyscalculia assessments in two different samples of CwD were used to assess the robustness of dyscalculia subtypes across measurement instruments.

To summarize, several studies have assessed subtypes of dyscalculia. However, results vary across studies, possibly due to the narrow range of skills assessed and different diagnostic tests used. The present study pursues the following questions: (1) Which subtypes in CwD can be identified by taking a broad range of mathematical skills (BNP, CNP, and calculation) and more general cognitive skills (attention, intelligence, reading skills, working memory) into account? (2) Is the identified pattern of dyscalculia subtypes robust? (3) Are there different subtypes in CwD that are related to specific comorbidity profiles?

In this study, the research questions outlined above were not analyzed assuming predefined (comorbid) groups. Rather, this study analyzed CwD (percentile rank <10 in standardized math assessments) and used a data-driven approach to identify subtypes. In summary, an exploratory approach was used to check whether subtypes of CwD which are characterized by comorbid cognitive profiles could be identified. To take the high comorbidity of dyscalculia and reading disorder into account, we also checked whether children with a comorbid reading disorder could be assigned to a specific subtype of CwD.

## Materials and Methods

### Sample

The total sample consisted of 174 CwD (mathematical abilities: percentile rank (PR) < 10; IQ > 70; level of education: grade 2, 3, and 4). The sample was part of a large-scale investigation of mathematical skills comprising 1,211 elementary school children. Data were collected in two separate contexts with partly different tests: 103 children (age: *M* = 8.94 years, *SD* = 1.05; 78 girls, 25 boys; grade 2: 34 students, grade 3: 48 students, grade 4: 21 students) were identified with a math test focusing on basic numerical abilities (ZAREKI-R; von Aster et al., 2006; in the following: ZAREKI-R sample). This subsample was recruited based on newspaper articles addressing families with (suspected) CwD. Between fall 2012 and fall 2013, these participants were invited to university, where testing took place in individual settings on two different days. A second sample of 71 children (age: *M* = 9.28 years, *SD* = 0.94; 46 girls, 25 boys; grade 2: 17 students, grade 3: 35 students, grade 4: 19 students) were classified with a math test mainly focusing on arithmetic skills (HRT 1–4; Haffner et al., 2005; in the following: HRT sample), which was administered in group settings taking 3 school hours in spring to fall 2013. The study was approved by the local ethics committee. To identify children with reading disorder (PR < 10), reading fluency was measured using the *Salzburger Lese-Screening* (SLS 1–4; Mayringer and Wimmer, 2003). The ZAREKI-R sample included 26 CwD with comorbid reading disorder, the HRT sample included 41 CwD with comorbid reading disorder.

### Tests

#### Diagnostic Tests: HRT 1–4 and ZAREKI-R

##### ZAREKI-R

The Neuropsychological Test Battery for Number Processing and Calculation in Children (*Neuropsychologische Testbatterie für Zahlenverarbeitung, und Rechnen bei Kindern*, ZAREKI-R; internal consistency between α = 0.93 and α = 0.97; von Aster et al., 2006) is a neuropsychological test battery that taps basic mathematical abilities ranging from counting, transcoding, magnitude and number line estimation to simple arithmetic and word problems. Theoretically, it is based on the Triple Code Model (Dehaene, 1992) and is often used for dyscalculia assessment in practice. Administration takes approximately 40 minutes. Compared to HRT 1–4, response modes are more versatile: Depending on the subtest, children either have to write down an answer, show something on a stimulus display or respond orally. In this study, the test was administered in a one-to-one setting in the facilities of Department of Psychology, University of Münster.

##### Heidelberg Calculation Test (HRT 1–4)

The Heidelberg calculation test (*Heidelberger Rechentest*; retest reliability: 0.93; Haffner et al., 2005) is a paper-pencil speed test of basic mathematical knowledge. HRT 1–4 consists of the two scales that are combined to a total score: (1) “arithmetic operations” (6 subtests: addition, subtraction, multiplication, division, fill-the-gap tasks, greater/less comparisons; retest reliability: 0.93) and (2) “numerical-logical and visual-spatial skills” (5 subtests: numerical series, lengths estimation, counting cubes, counting magnitudes, connecting numbers; retest reliability: 0.87; Haffner et al., 2005). T-score norms (i.e., a standardization resulting in a mean of 50 and standard deviation of 10) are available for every quarter of the school year. In this study, the test was administered in group setting, either in the facilities of Department of Psychology, University of Münster, or in classroom.

#### Intelligence

Different tests were used to assess intelligence. In the ZAREKI-R sample, the perceptual reasoning index (retest-reliability: 0.93) of the WISC-IV (Wechsler, 2011) was used to assess intelligence. In the HRT sample, intelligence was measured using the language-free group test CFT 1-R with a retest-reliability of 0.95 (Weiß and Osterland, 2013). Both tests focus on fluid intelligence and do not require any language skills.

#### Reading Fluency

To measure reading fluency, the *Salzburger Lese-Screening* (SLS 1–4) with a parallel test reliability of at least 0.90 was used (Mayringer and Wimmer, 2003): Children had to read as many simple and unambiguous sentences (e.g., Bananas are blue) as possible within 3 minutes; By ticking a box at the end of each sentence, children had to specify if the sentences were correct or incorrect, and the more correct answers a child gave, the higher the reading fluency (Mayringer and Wimmer, 2003).

#### Working Memory

The task *matrix span* included in the CODY-M 2–4 battery with a retest reliability of 0.61 (Kuhn et al., 2017) was used in both samples to test the visual-spatial working memory. During this test, children had to memorize a pattern of dots and they had to solve a distracting task; afterward, they had to reproduce the dot pattern (Raddatz et al., 2017). In addition, the *verbal span* test (reported reliability: α = 0.78) of the working memory scales by Vock and Holling (2008) was used in the ZAREKI-R sample: First, participants had to remember a list of words; next, a distracting classification task was presented, after which the initially learned word had to be retrieved. Raw scores of the verbal span task were transformed to standardized T-scores based on the total sample. In the ZAREKI-R sample, mean working memory performance was calculated based on both working memory tasks.

#### Mathematical Abilities

Mathematical abilities were assessed using the CODY-M 2–4 battery (Kuhn et al., 2017). According to the CODY-M 2–4 manual, subscale scores for (1) BNP (retest reliability: 0.72), (2) CNP (retest reliability: 0.76), and (3) Calculation skills (retest reliability: 0.85) were computed to measure different components of mathematical skills (Kuhn et al., 2017). All following descriptions of the mathematical tests are based on Raddatz et al. (2017).

##### Basic Numerical Processing (BNP)

Efficiency in counting was tested by *dot enumeration*: 1–9 black dots had to be counted as quickly and correctly as possible. Across all correct responses, the median of the children’s reaction times was computed. In addition, BNP was tested by two comparison tasks (c.f. Defever et al., 2013): Two different Arabic numerals (*symbolic magnitude comparison*) or a numerosity of dots on one side and an Arabic numeral on the other side (*mixed magnitude comparison*) were displayed on a screen, and children had to decide which of the shown entities was larger (right or left). These tasks have in common that they all assess core number competencies and incorporate very simple and basic tasks as enumeration tasks and the comparison of magnitudes (Reeve et al., 2012; Kuhn et al., 2017).

##### Complex Number Processing (CNP)

One task tests the precision of the mental *number line* (based on Siegler and Booth, 2004): A number was shown on a screen and the children had to locate this number with a computer mouse on an unscaled number line (only the endpoints were labeled with *0* and *100*). The *number sets* task (based on Geary et al., 2009) was used to assess the efficiency of number processing across presentation formats: Again, an Arabic numeral was shown at the top of the screen. In addition, numbers and/or geometric figures (= a number set) were shown at the bottom of the screen. Children had to compare the sum of the elements represented as a number set with the number above and they had to decide whether the sum of the number set was equal to the shown number above. Two target numbers were used (5 and 9) in this speed test. For example, on the top of the screen a 5 (as an Arabic numeral) was shown as the target number. At the bottom of the screen, three geometric figures and a 1 (as an Arabic numeral) were shown. In this example, the child had to calculate *3 (geometric figures) + 1 (as an Arabic numeral) = 4*, and compare the 4 (the sum of the number set) to the 5 (the target number) and check whether the number set is equal or unequal to the target number. *Transcoding* tasks assessed the ability to translate heard numbers (presented by headphones) into written Arabic numerals. These different tasks have in common that they assess mathematical precursor skills that require more *complex number processing* (CNP; Nuerk et al., 2006; Kuhn et al., 2017).

##### Calculation (CALC)

The participants had to solve tasks focusing on (1) *addition*, (2) *subtraction*, and (3) *multiplication mixed with place holder tasks*. The addition and subtraction tasks ranged from fact retrieval (e.g., 1 + 8) to more difficult tasks (e g., 183–18). Place holder tasks are arithmetic tasks that are not to be solved linearly from left to right, but an element of the equation has to be determined (e.g., 4 + × = 7; what is ×?). These tasks have in common that they all require the skill to perform arithmetic.

#### Attention

In the ZAREKI-R sample, three subtests of the KITAP were used to measure different aspects of attention (Zimmermann et al., 2005): (1) The subtest *alertness* (split-half reliability of the reaction time’s median: 0.96) tests the intensity of attention. Children had to react as quickly as possible to a witch appearing on a screen. (2) In the subtest *sustained attention* (split-half reliability of the reaction time’s median: 0.93), a sequence of ghosts briefly appeared in the windows of a castle and disappeared. Children had to check whether the ghost they saw was identical to the one seen just before. (3) The subtest *flexibility* (split-half reliability of the reaction time’s median: 0.93) was used to measure selective attention, i.e., the ability to adapt the focus of attention. The screen was split in two and on each side an identically shaped stimulus (dragon) that varied in color appeared (one stimulus was blue, one was green). The target (= the color of the stimulus) changed alternately and children had to react to where the target color appeared (on the left/right side) as quickly and correctly as possible by pushing a button. The standardized mean of these three attention tests was calculated in order to obtain a score for attention. All descriptions of the used tests to measure attention are based on the KITAP manual (Zimmermann et al., 2005). In the sample measured at school (HRT 1–4 as dyscalculia criterion), no attention data could be captured as the testing procedure requires individual settings.

### Statistical Analyses

All calculations were carried out with version 4.0.0 of the statistical software R (R Core Team, 2020). The values of all variables were T-standardized resulting in T-scores; i.e., the standardization sample had a mean of 50 and a standard deviation of 10. Necessary data transformations were carried out using the R-package dplyr (Singh and Soman, 2019).

To identify subtypes of dyscalculia, model-based clustering (parameterized finite Gaussian mixture models) based on the R-package mclust (Fraley et al., 2020) was performed. Each participant of a sample was assigned to a single cluster by calculating the probability of a person belonging to a specific cluster based on the individual cognitive profile (Vanbinst et al., 2015). All participants assigned to the same cluster can be interpreted as a subgroup, and the number of clusters corresponds to the number of subgroups (Bouveyron et al., 2019).

The number of clusters was determined based on the Bayesian Information Criterion (BIC; Bouveyron et al., 2019). Different (preset) competing models that can plausibly describe cluster structures were used to determine the number of clusters that fits the data best: These models vary in their assumptions regarding the geometric characteristics of the clusters as their spatial orientation or their volume (equal vs. varying volume), for example (Makhabel et al., 2017; Bouveyron et al., 2019; Fraley et al., 2020). For each possible model, the BIC is calculated for different numbers of clusters, and the lowest absolute BIC of a model-cluster-combination suggests that this solution fits the data best (Vanbinst et al., 2015; Bouveyron et al., 2019). Each of the possible model-cluster-combinations was compared to other possible model-cluster-combinations in order to find the model-cluster-combination with the strongest evidence. Each model has a specific identifier (e.g., “EEI”) and the clustering procedure automatically chooses the most appropriate out of different models. The identifier can be used to look up the characteristics of this model in the manual of the mclust-package: For example, the identifier EEI means that there are diagonal clusters with equal volume and equal shape (Fraley et al., 2020). So, if a specific model-cluster-combination of the EEI model, for example, has the lowest absolute BIC, this means that this model-cluster-combination is the best solution with regard to the data. As in other studies with similar approaches (e.g., Vanbinst et al., 2015), the results of the model-cluster-combination with the lowest absolute BIC are presented. It makes sense to only describe and interpret this model-cluster-combination, since all other model-cluster-combinations fit the empirical data less well and therefore there is no convincing evidence for these other solutions.

To check whether the subgroup-solution of the clustering process is robust, two different ways of clustering were used. First, a clustering was carried out at the *construct level* as described before [intelligence, reading fluency, working memory, Basic numerical processing (BNP), Complex number processing (CNP), Calculation (CALC), and Attention]. Further, another model-based clustering used data at the *subtest level* (variables: intelligence, reading fluency, matrix span, verbal span, enumeration, symbolic magnitude comparison, mixed magnitude comparison, number line, number sets, transcoding, addition, subtraction, multiplication mixed with place holder tasks, attention). If both clustering approaches lead to similar or identical solutions, this is an indication for the validity of the superordinate constructs and for the robustness of the results across levels of measurement.

The resulting subgroups of the best-fitting model were then compared with regard to each construct/subtest used to cluster these subgroups. These comparisons were based on using frequentist and Bayesian *t*-tests to check the differences and similarities of the subgroups in detail. The significance level for the frequentist *t*-tests was adjusted by the *sequentially rejective Bonferroni test* to prevent the alpha error from accumulating (Holm, 1979), and Cohen’s *d* as an effect size for between-group differences was computed with the R-package lsr (Navarro, 2015). Bayesian *t*-tests were carried out to check the robustness of the frequentist results: Both approaches can lead to different conclusions, but if the results of frequentist analyses and the results of Bayesian analyses point to the same direction, they can be rated as robust (Lindley, 1957; Sprenger, 2013; Wagenmakers et al., 2018). In contrast to frequentist statistics, Bayesian methods (e.g., Bayesian *t*-tests) cannot only unravel whether there is evidence for a difference between groups, but also verify that there is evidence for equality of the analyzed groups (Rouder et al., 2012; Wagenmakers et al., 2018). Bayesian analyses were conducted with the R-package BayesFactor (Morey et al., 2018). An important difference between frequentist and Bayesian statistics is that Bayesian statistics do not provide *p*-values, but Bayes Factors (BF). A BF lower than 0.33 suggests moderate evidence for the null hypothesis, a BF lower than 0.10 suggests strong evidence for the null hypothesis and a BF lower than 0.033 suggests very strong evidence for the null hypothesis; the other way round, a BF above three suggests moderate evidence for the alternative hypothesis, a BF above 10 suggests strong evidence for the alternative hypothesis and a BF above 30 suggests very strong evidence for the alternative hypothesis (Wagenmakers et al., 2018).

In addition, repeated-measures ANOVAs were calculated to check whether there was a main effect of subgroup (between-group factor), i.e., a mean difference across constructs/subtests between the assumed subtypes (Bulut and Desjardins, 2018; Bulut and Desjardins, 2020). Further, we also checked whether there was a main effect of test (within-group factor), i.e., whether mean performance across constructs/subtests varied independently of subgroups (within groups: intelligence, reading fluency, working memory, BNP, CNP, calculation, and attention). Most importantly, we investigated interaction effects to check whether the identified subgroups differed disproportionately with regard to each different construct/subtest. If subgroups differ disproportionately, profile lines of the subgroups do not run in parallel. Parallelism was additionally tested using profile analysis based on the R package profileR (Bulut and Desjardins, 2018, 2020). Necessary data set modifications were done by using the R-package reshape2 (Wickham, 2020) and ANOVAs as well as effect sizes for ANOVAs – generalized eta squared ($\eta_{G}^{2}$; Bakeman, 2005) – were computed with the R-package ez (Lawrence, 2016).

As already indicated, it was checked whether relative frequency of children with dyscalculia and a comorbid reading disorder differed across subtypes. To test this, *χ*^2^-tests were conducted with the categorical variables (1) reading disorder (yes/no) and (2) subtype. If the prerequisites for *χ*^2^-tests (i.e., sufficient cell sample sizes) were not met, Fisher’s exact test for count data was conducted.

Missing data can significantly influence and distort the results of statistical analyses. Therefore, a two-step approach was used here. In a first step, only complete data sets (data of children with no missing data) were analyzed. In this case, the ZAREKI-R sample consisted of 93 children (26 children with a comorbid reading disorder) and the HRT sample consisted of 67 children (38 children with a comorbid reading disorder). In a second step, the function imputeData from the R-package mclust (Fraley et al., 2020) was used to impute missing data, and the most important calculations were repeated to check the robustness of the results. Because added data vary as a function of random start points, it is strongly recommended to compute multiple imputations (Fraley et al., 2012). To check if the results were robust across imputations, central calculations were rerun with imputed data sets generated with three random seeds (3; 3,000; 3,000,000) (Fraley et al., 2012). If all results point into the same direction, the results can be interpreted as robust.

## Results

In all cases (ZAREKI-R sample and HRT sample; analyses on construct level and on subtest level; with and without imputation), mixture model analyses consistently suggested that there were two subgroups of CwD. The EEI-model (cluster characteristics: two diagonal clusters with equal volume and equal shape; Fraley et al., 2020, p. 105) was the model that described the data best in both samples (ZAREKI-R and HRT; each without imputations and clustered by constructs). The absolute BIC of the ZAREKI-R sample (without imputation and with analyses on construct level) was 4,390 and the absolute BIC of the HRT sample (without imputation and with analyses on construct level) was 2,851. The results described in the following are based on complete data sets at the construct level. If deviations occurred in alternative calculations (with imputed data or at the subtest level), these deviations are reported.

For both resulting subgroups of the ZAREKI-R sample, the results of descriptive analyses (mean, standard deviation, skewness, kurtosis, minimum, and maximum) for complete data sets (construct level) are shown in Table 1. The results of descriptive analyses of the HRT sample for complete data sets (construct level) are shown in Table 2. To check the robustness of the results, the clustering processes were carried out again at the level of subtests: The results of the descriptive analyses for the resulting subgroups are shown in Table 3 (ZAREKI-R sample, complete data sets) and Table 4 (HRT sample, complete data sets).

**TABLE 1:** Descriptive statistics of the ZAREKI-R sample—clustered by constructs.

	Subgroup 1 (*n* = 27)						Subgroup 2 (*n* = 66)					
	*M*	*SD*	Skewness	Kurtosis	Min.	Max.	*M*	*SD*	Skewness	Kurtosis	Min.	Max.
Intelligence	48.24	6.09	0.32	1.76	39.17	58.33	45.34	6.01	0.02	2.41	30.83	58.33
Reading fluency	45.80	11.08	0.15	2.25	24.67	68.00	41.51	9.59	0.54	4.01	21.33	72.00
Working Memory	47.30	5.77	0.32	2.48	37.50	60.50	45.87	4.92	0.20	3.61	33.00	59.50
Basic numerical processing	50.89	7.73	0.40	2.94	36.00	67.00	43.38	6.76	0.31	2.92	29.00	60.00
Complex number processing	47.41	5.06	−0.11	1.73	38.00	55.00	40.64	4.83	−0.24	2.56	30.00	51.00
Calculation	47.48	4.11	0.02	3.91	37.00	58.00	37.30	3.95	0.11	2.30	30.00	46.00
Attention	47.53	7.22	−0.15	1.74	34.67	57.67	41.55	7.48	−0.06	2.45	22.33	58.00

*All variables are T-standardized (mean = 50, SD = 10).*

**TABLE 2:** Descriptive statistics of the HRT sample—clustered by constructs.

	Subgroup 1 (*n* = 39)						Subgroup 2 (*n* = 28)					
	*M*	*SD*	Skewness	Kurtosis	Min.	Max.	*M*	*SD*	Skewness	Kurtosis	Min.	Max.
Intelligence	47.35	9.32	0.32	2.48	31.33	67.00	42.17	6.68	−0.05	2.61	30.00	55.33
Reading fluency	38.17	9.97	0.15	2.32	20.00	60.00	33.79	10.24	0.67	2.76	18.67	58.00
Working Memory	45.28	9.12	0.21	2.13	32.00	65.00	43.82	8.33	0.14	2.74	26.00	60.00
Basic numerical processing	46.33	8.37	0.64	3.64	31.00	72.00	46.11	9.08	−0.03	2.55	28.00	65.00
Complex number processing	48.03	4.05	0.11	2.79	39.00	58.00	36.79	4.23	−0.29	2.66	28.00	44.00
Calculation	44.77	4.42	0.04	2.06	37.00	53.00	35.82	4.23	−0.13	3.62	26.00	46.00

*All variables are T-standardized (mean = 50, SD = 10).*

**TABLE 3:** Descriptive statistics of the ZAREKI-R sample–clustered by subtests.

	Subgroup 1 (*n* = 26)						Subgroup 2 (*n* = 60)					
	*M*	*SD*	Skewness	Kurtosis	Mix.	Max.	*M*	*SD*	Skewness	Kurtosis	Min.	Max.
Intelligence	46.99	6.36	0.47	2.00	38.33	58.33	45.72	5.88	0.08	2.58	30.83	58.33
Reading fluency	45.49	10.32	0.51	2.37	28.67	68.00	40.88	9.37	0.36	4.03	21.33	72.00
Matrix span	46.23	8.82	0.14	2.38	33.00	65.00	45.87	7.13	0.02	2.77	30.00	61.00
Verbal span	48.54	5.43	0.50	2.71	41.00	61.00	45.98	6.32	0.97	3.97	36.00	67.00
Dot enumeration	51.35	10.83	0.52	2.70	33.00	75.00	42.22	8.53	−0.20	3.12	22.00	65.00
Symbolic magnitude comparison	50.15	9.56	0.10	2.11	35.00	68.00	43.77	11.96	0.31	2.40	22.00	69.00
Mixed magnitude comparison	51.77	13.14	0.12	2.15	31.00	75.00	44.02	10.95	0.16	2.56	22.00	71.00
Transcoding	46.73	8.30	−0.38	1.65	32.00	58.00	41.03	9.14	−0.04	1.84	22.00	54.00
Number sets	47.35	8.67	0.49	2.99	32.00	68.00	41.02	6.30	0.18	2.68	28.00	57.00
Number line	48.00	5.58	−0.36	2.06	37.00	57.00	40.30	7.63	0.41	3.48	23.00	63.00
Addition	48.35	6.39	0.42	2.31	39.00	61.00	36.47	4.73	−0.27	2.86	24.00	46.00
Subtraction	44.96	4.89	0.31	2.91	37.00	57.00	37.27	6.20	0.52	2.28	26.00	49.00
Multiplication mixed with place holder tasks	48.62	7.51	−0.10	2.96	33.00	65.00	38.35	7.04	0.13	2.35	25.00	53.00
Attention	47.24	7.53	−0.29	2.00	32.00	57.67	41.68	7.76	−0.04	2.42	22.33	58.00

*All variables are T-standardized (mean = 50, SD = 10).*

**TABLE 4:** Descriptive statistics of the HRT 1-4 sample—clustered by subtests.

	Subgroup 1 (*n* = 43)						Subgroup 2 (*n* = 24)					
	*M*	*SD*	Skewness	Kurtosis	Min.	Max.	*M*	*SD*	Skewness	Kurtosis	Min.	Max.
Intelligence	46.40	9.45	0.39	2.53	30.00	67.00	43.01	6.63	−0.16	2.61	30.00	55.33
Reading fluency	38.03	10.20	0.05	2.16	20.00	60.00	33.31	9.80	0.92	3.60	18.67	58.00
Matrix span	45.07	9.08	0.24	2.10	32.00	65.00	43.96	8.30	0.08	2.93	26.00	60.00
Dot enumeration	46.70	11.31	0.65	3.18	22.00	74.00	42.25	9.66	−0.03	1.88	27.00	58.00
Symbolic magnitude comparison	47.58	11.90	0.74	2.79	29.00	75.00	44.63	12.46	−0.02	2.12	22.00	65.00
Mixed magnitude comparison	46.88	12.44	0.40	2.86	22.00	75.00	47.42	11.50	0.03	2.11	26.00	69.00
Transcoding	49.91	5.72	−0.58	2.16	40.00	58.00	34.63	6.21	−0.11	2.13	22.00	44.00
Number sets	44.56	7.18	0.11	2.74	29.00	60.00	37.96	6.08	−0.13	2.11	26.00	49.00
Number line	48.00	8.12	0.57	2.85	36.00	69.00	35.75	4.61	0.96	4.19	29.00	49.00
Addition	44.70	6.70	0.58	2.90	34.00	60.00	35.04	6.54	0.30	2.97	24.00	51.00
Subtraction	43.07	6.39	0.79	4.62	30.00	65.00	34.67	5.35	−0.33	2.17	26.00	43.00
Multiplication mixed with place holder tasks	45.28	7.78	0.29	2.85	30.00	64.00	35.46	5.64	−0.52	2.81	24.00	45.00

*All variables are T-standardized (mean = 50, SD = 10).*

Mean comparisons of the two identified subgroups for complete data sets are shown in Table 5 (construct level) and Table 6 (subtest level). In each sample, there was one subgroup (named: subgroup 2) that almost always reached lower test scores (means) in comparison to the other subgroup (named: subgroup 1). Differences between the two subgroups were very small for some measures (e.g., for BNP in the HRT sample or for working memory in both samples), and significantly large for others (e.g., CNP and CALC in both samples). The descriptive analyses therefore suggest that the profiles of the subgroups differed, and that the distinctiveness of subgroups’ profiles varied between cognitive measures.

**TABLE 5:** Subgroup mean comparison—clustered by constructs.

	ZAREKI-R			HRT 1–4		
	*t*-test	Cohen’s *d*	BF	*t*-test	Cohen’s *d*	BF
Intelligence	2.10*	0.48	1.57	2.65*	0.62	3.48
Reading fluency	1.87	0.43	1.07	1.76	0.43	0.93
Working memory	1.20	0.28	0.44	0.67	0.17	0.31
Basic numerical processing	4.66***	1.07	1589.59	0.11	0.03	0.25
Complex number processing	6.06***	1.38	3.28×10^5^	11.00***	2.73	2.15×10^13^
Calculation	11.16***	2.55	3.19×10^15^	8.32***	2.06	7.63×10^8^
Attention	3.54***	0.81	43.98	−	−	−

Interpretation of p-values: p < 0.05*, p < 0.01**, p < 0.001*** the significance level was adjusted by the sequentially rejective Bonferroni test to prevent the alpha error from accumulating (Holm, 1979); interpretation of BFs (Wagenmakers et al., 2018): BF < 0.33 (moderate evidence for the null hypothesis), BF < .10 (strong evidence for the null hypothesis), BF < 0.033 (very strong evidence for the null hypothesis), BF > 3 (moderate evidence for the alternative hypothesis), BF > 10 (strong evidence for the alternative hypothesis), BF > 30 (very strong evidence for the alternative hypothesis).

**TABLE 6:** Subgroup mean comparison—clustered by subtests.

	ZAREKI-R			HRT 1–4		
	*t*-test	Cohen’s *d*	BF	*t*-test	Cohen’s *d*	BF
Intelligence	0.89	0.21	0.34	1.55	0.39	0.71
Reading fluency	2.03*	0.48	1.40	1.84	0.47	1.07
Matrix span	0.20	0.05	0.25	0.49	0.13	0.29
Verbal span	1.79	0.42	0.95	−	−	−
Dot enumeration	4.19***	0.98	306.95	1.62	0.41	0.78
Symbolic magnitude comparison	2.41	0.57	2.81	0.96	0.24	0.38
Mixed magnitude comparison	2.84*	0.67	7.09	−0.17	0.04	0.26
Transcoding	2.73*	0.64	5.53	10.17***	2.59	9.11×10^11^
Number sets	3.80**	0.89	92.25	3.80**	0.97	81.06
Number line	4.63***	1.09	1307.06	7.88***	1.73	2.12×10^6^
Addition	9.58***	2.25	1.05×10^12^	5.71***	1.45	3.86×10^4^
Subtraction	5.61***	1.32	4.69×10^4^	5.46***	1.39	1.62×10^4^
Multiplication mixed with place holder tasks	6.09***	1.43	3.07×10^5^	5.43***	1.38	1.45×10^4^
Attention	3.08*	0.72	12.77	−	−	−

Interpretation of p-values: p < 0.05*, p < 0.01**, p < 0.001***; the significance level was adjusted by the sequentially rejective Bonferroni test to prevent the alpha error from accumulating (Holm, 1979); interpretation of BFs (Wagenmakers et al., 2018): BF < 0.33 (moderate evidence for the null hypothesis), BF < 0.10 (strong evidence for the null hypothesis), BF < 0.033 (very strong evidence for the null hypothesis), BF > 3 (moderate evidence for the alternative hypothesis), BF > 10 (strong evidence for the alternative hypothesis), BF > 30 (very strong evidence for the alternative hypothesis).

This was tested by ANOVA, showing for the HRT sample that (a) there was a significant main effect of the factor subgroup, *F*_(__1, 65)_ = 37.84, *p* < 0.001, $\eta_{G}^{2}$ = 0.10; (b) there was a significant main effect for the different constructs, *F*_(__5, 325)_ = 15.92, *p* < 0.001, $\eta_{G}^{2}$ = 0.16; (c) there was a significant interaction effect for subgroups and constructs, *F*_(__5, 325)_ = 5.08, *p* < 0.001, $\eta_{G}^{2}$ = 0.06. The profile analysis confirmed these results, providing evidence against parallelism of the subgroups‘ profiles, *F*_(__5, 61)_ = 8.54, *p* < 0.001.

In line with these findings, an ANOVA for the ZAREKI-R sample also showed (a) a significant main effect for subgroups, *F*_(__1, 91)_ = 77.03, *p* < 0.001, $\eta_{G}^{2}$ = 0.13; (b) a significant main effect for the different constructs, *F*_(__6, 546)_ = 6.04, *p* < 0.001, $\eta_{G}^{2}$ = 0.05; (c) a significant interaction effect for subgroups and constructs, *F*_(__6, 546)_ = 3.96, *p* < 0.001, $\eta_{G}^{2}$ = 0.03. Again, profile analysis indicated that there was no parallelism of the subgroups’ profiles, *F*_(__6, 86)_ = 7.12, *p* < 0.001. The next paragraphs describe these profile differences in more detail.

### Intelligence

Means for intelligence in subgroup 1 were higher than means for intelligence in subgroup 2 across samples and for all ways of clustering. This difference was significant in the main analyses if the subgroups were clustered at the construct level (Table 5), but not robust in all *t*-tests with imputations. In some cases, the results of Bayesian analyses did not confirm the significant results of the frequentist analyses, e.g., ZAREKI-R without imputations (Table 5): *t*(91) = 2.10, *p* < 0.05, but BF = 1.57. Differences in intelligence were not significant if the subgroups were clustered at the subtest level (Table 6). Overall, the data suggest a very small difference between subgroups in terms of their language-free intelligence (subgroup 1 > subgroup 2).

### Reading Fluency

Although means in subgroup 1 were generally higher than means in subgroup 2, there was no clear statistical evidence for differences between subgroups. In the ZAREKI-R sample, there was a significant difference between the two subgroups if imputations were used [seed = 3,000,000; *t*(101) = 2.53, *p* < 0.05, BF = 3.52], but this difference was not robust. Overall, subgroup differences seemed to be very small and mostly insignificant, but there was also no clear Bayesian evidence for the groups being equal.

### Working Memory

Working memory differences between subgroups were very small—both at construct level and at the subtest level: The *t*-tests were not significant and the BFs were below 1. So, there was no evidence for a difference between these two groups with regard to working memory and there was even moderate evidence for the null hypothesis, i.e., equality of subgroups (HRT sample without imputations: BF = 0.31). In the HRT sample, only the matrix span task was used to assess working memory and these results were identical if the subgroups were clustered by subtests (Table 6): In many cases, there was even moderate evidence for the subgroups being equal because BFs were below 0.33. It should be kept in mind here that in the ZAREKI-R sample, two different tests for assessing working memory were used: matrix span and verbal span (cf. section “Working Memory”). If the subgroups were clustered by subtests, there were significant differences (based on imputed data), but these differences were not robust.

### Mathematical Skills

The two subgroups in both samples differed very strongly in their mathematical skills; especially for CALC and CNP, very strong and robust evidence for a difference was found. In addition, a significant difference between the two subgroups occurred in the ZAREKI-R sample for BNP, *t*(91) = 4.66, *p* < 0.001, BF = 1589.59. However, there was no such difference in the HRT sample, *t*(65) = 0.11, *p* = 0.92, BF = 0.25. The results were robust. Of all constructs or subtests, subgroups differed most strongly in terms of their mathematical skills.

### Attention

Attention was only assessed in the ZAREKI-R sample, and the scores for attention were higher in subgroup 1 than in subgroup 2. There was a significant difference between the two subgroups in this sample if clustered by constructs, *t*(91) = 3.54, *p* < 0.001, BF = 43.98. If clustered by subtests, this difference was significant as well, *t*(84) = 3.08, *p* < 0.05, BF = 12.77. These results were robust if imputations were used. There was clear evidence for a difference between the subgroups, but this difference was not as pronounced as for the mathematical skills (CALC and CNP).

### Comorbid Reading Disorder

*χ*^2^-tests (for complete data sets at the construct level) showed no significant associations between reading disorder (PR < 10) and the identified subtypes (HRT: *χ*^2^ = 0.66, *p* = 0.42; ZAREKI-R: *χ*^2^ < 0.01, *p* = 0.98). For alternative calculations (e.g., analyses at the subtest level), the results were almost identical and therefore robust.

### Comorbid Low Intelligence

Overall, the model-based clustering revealed a slightly impaired subgroup on the one side (subgroup 1) and a severely impaired subgroup on the other side (subgroup 2) in CwD (mathematical abilities: PR < 10). The fact that subgroup 2 showed lower performance in nearly all areas might lead to the assumption that this might be due to a substantial proportion of children with low intelligence (PR < 10) in this subgroup. However, in the ZAREKI-R sample, Fisher’s exact test for count data did not suggest a systematic dependency between low intelligence and subgroup affiliation, *p* = 0.32. In the HRT sample, there again was no systematic dependency between low intelligence and subgroup affiliation, *χ*^2^ = 0.36, *p* = 0.55. Results appeared robust across alternative calculations.

### Comorbid Attention Deficits

There was a significant difference in attention between the two subgroups in the ZAREKI-R sample (subgroup 1 > subgroup 2). In fact, 22 of 66 children of subgroup 2 and only 3 of 27 children of subgroup 1 displayed deficits in attention (PR < 10). Fisher’s exact test for count data suggested a systematic dependency between attention deficits and subgroup affiliation (*p* = 0.038). Again, alternative calculations provided similar results and were therefore robust.

## Discussion

In contrast to many other studies with data-driven designs (e.g., Bartelet et al., 2014; Chan and Wong, 2020), no large number of subtypes in CwD was identified in this methodically advanced study. Two subgroups of children with dyscalculia were consistently found, and this result was robust regardless of whether (a) only complete data sets or imputed data sets were used, (b) the clustering was carried out at the level of subtests (e.g., dot enumeration, magnitude comparison) or aggregated constructs (e.g., basic numerical processing) or (c) different dyscalculia assessments were used (HRT 1–4 or ZAREKI-R). In addition, the number of subgroups was not affected by taking the construct of attention into account (only assessed in the ZAREKI-R sample). Although not a complete multiverse analysis (Steegen et al., 2016), the results suggest very convincingly that there are two subtypes of CwD: a slightly impaired subtype (subgroup 1) and a severely impaired group (subgroup 2).

The results of this study underline that different study designs and clustering methods can come to different results. However, this does not necessarily imply that other ways to cluster CwD are misguided: Of course, the formation of subtypes is always a generalization and therefore just a heuristic to facilitate practical decision-making. It must always be weighed to which extent individualization or generalization serves a specific purpose. Furthermore, subtypes from data-based studies depend on the sample, assessments, and constructs under investigation. Hence, results of the present study may differ from other studies (e.g., Bartelet et al., 2014; Chan and Wong, 2020) because only children with very poor mathematical performance (PR < 10) were examined here, and children at risk of dyscalculia (PR between 10 and 25) were excluded. However, children at risk of dyscalculia may display very heterogeneous deficits, and hence, excluding this group may explain the comparably small number of subgroups in our study. Further, the relatively small sample size for a mixture model analysis may be regarded as a key limitation—more subgroups might have emerged in the case of larger sample sizes of CwD, which, however, are resource-intensive to obtain.

Results of the ZAREKI-R sample differed from the HRT sample in one aspect: There was a large difference in basic numerical processing between the two subgroups in the ZAREKI-R sample, but there was no such difference in the HRT sample. This could be a bias due to the different focus of these diagnostic tests: The HRT 1–4 mainly tests the ability to calculate and arithmetic skills, but the HRT 1–4 does not strongly focus on BNP, whereas the ZAREKI-R does. Nevertheless, the two subgroups – regardless of the diagnostic test – showed large differences in calculation and complex number processing. All in all, subgroups differed in particular in the extent to which their mathematical skills were impaired. The fact that CwD can be divided into subgroups based on the severity of impairments is in line with other studies that have also distinguished children with mathematical deficits into subgroups based on their mathematical skills (e.g., Skagerlund and Träff, 2016).

The results of this study suggest that working memory (in particular if measured with a matrix span task that is combined with a distracting task) and reading fluency do not appear to be helpful to characterize different subtypes. The result that reading ability seems to be of no importance for the characterization of subgroups contradicts some prior findings of subtyping CwD (e.g., Ozols and Rourke, 1988; Rourke, 1993). This result also seems to contradict the fact that dyscalculic children with comorbid reading disorders are usually more impaired than children with isolated dyscalculia (e.g., Kißler et al., 2021). Hence, finding a more impaired subtype of CwD with comorbid reading disorders would have been plausible. However, our analysis strategy did not provide results that support the view that reading difficulties co-occur with more severe mathematical difficulties, possibly due to the relatively strict criterion for identifying children with dyscalculia in this study (PR < 10), which results in lower comorbidity rates between dyscalculia and reading disorders (Moll et al., 2014). Even though our analyzes could not find a subtype that is characterized by comorbid reading disorders, remedial teaching should nevertheless be individually tailored to the respective child and the child’s needs.

In this study, intelligence seems barely relevant to subtype CwD–in contrast to the findings of Bartelet et al. (2014), for example. Overall, we found a slightly impaired subtype (subgroup 1) as well as a severely impaired subtype (subgroup 2) in CwD, and the intelligence of subtype 2 tends to be slightly lower. Even if there seems to be a small difference in terms of intelligence between the subtypes, no significant accumulation of children with an IQ below 80 (PR < 10) in subtype 2 could be found. Nevertheless, the fact that the children in subtype 2 showed a generally lower cognitive profile could be partly linked to a lower intelligence. Overall, in this study, intelligence seemed to be less relevant in the formation of subgroups compared to attention and math skills themselves—this supports the assumption that the intelligence discrepancy criterion should be of secondary importance (e.g., Ehlert et al., 2012; Kuhn et al., 2013).

In the ZAREKI-R sample, there was strong evidence that attention matters: Subgroup 2 was severely impaired in attention. Due to the study design, it is unclear whether this finding depends on the selected test method (ZAREKI-R) and setting (individual administration at university) or whether this is a general characteristic. However, comorbidity in terms of attention deficits appears to be relevant in the characterization of subtypes in CwD: There seems to be one subtype of CwD that does not only display major difficulties in mathematics but is also characterized by considerable deficits in attention. Because this result was only obtained in one of the two subsamples analyzed here, more research is needed to replicate this result.

The fact that the subtypes differ most strongly in terms of their mathematical skills means that among CwD (PR < 10), there is one group of children that is even more strongly impaired. There is no subtype that is characterized in particular by comorbid deficits in single non-mathematical abilities (e.g., working memory or reading skills). Rather, the subtypes tend to differ in more than one area (but in particular in different mathematical abilities and in attention at once), meaning that comorbidity is still a relevant issue when talking about subtypes in dyscalculia. Overall, the existence of subtype 2 allows the conclusion that attention problems are present in children with severe impairments in their mathematical abilities.

The results of this data-driven study suggest that the existence of two subtypes is robust and plausible. From a practical point of view, a two-subtype-solution can be useful for educational decision-making: Even though each child with dyscalculia needs specific intervention approaches and materials tailored to its individual needs, this two-subtype solution could be the basis for the development of different educational materials taking into account the two broad subtypes that were found in this study. Specifically, when planning interventions to foster CwD, it is important to have in mind that some of these children have substantial attention problems. In further studies, it should be examined whether interventions to improve attention, or taking attention deficits systematically into account during intervention, could lead to an improvement in mathematical skills in this subtype. In the research field of training programs, there are first approaches that try to take the comorbidity of reading disorders and attention disorders into account (Koenigs et al., 2019). Similar approaches for CwD would make sense in light of the results of this study. For educational practice, this means that math teachers should be made aware that children with the biggest problems in mathematics tend to have problems in attention too—and these children may have to be separately addressed. However, recent research (von Wirth et al., 2021) suggests that attention deficits in children with ADHD do not substantially affect basic numerical processing, and that ADHD in children with dyscalculia does not substantially deteriorate mathematical deficits. Therefore, further research is needed to illuminate the role of attention and attention deficits in children with dyscalculia.

## Data Availability Statement

The raw data supporting the conclusions of this article will be made available by the authors, without undue reservation.

## Ethics Statement

The studies involving human participants were reviewed and approved by the Ethikkommission des Fachbereichs 7, Psychologie und Sportwissenschaft, Westfälische Wilhelms-Universität Münster (ethics committee of Faculty 7 - Psychology & Sports Sciences, University of Münster). Written informed consent to participate in this study was provided by the participants’ legal guardian/next of kin.

## Author Contributions

J-TK: idea for writing this manuscript. J-TK and CS: data collection. CK: creating the basic structure of the draft and writing the draft of the manuscript. CK, J-TK, and CS: revisions to the text. CK and J-TK: search for articles. Note on the writing process of the method section: (a) CK: writing the draft of the chapter. (b) J-TK and CS: check and ensure that the methodological procedure is reported correctly. Note on the writing process of the results section: CK and J-TK: writing the R-script. CK and J-TK: creating the tables. All authors contributed to the article and approved the submitted version.

## Conflict of Interest

The authors declare that the research was conducted in the absence of any commercial or financial relationships that could be construed as a potential conflict of interest.
