# Supplementary material for: Two Dyscalculia Subtypes With Similar, Low Comorbidity Profiles: A Mixture Model Analysis
Source: Front Psychol. 2021 Jun 21;12:589506. doi: 10.3389/fpsyg.2021.589506 (PMC8255685; doi:10.3389/fpsyg.2021.589506)
Supplement: Supplementary file 1 [file Table_1.pdf]

Two dyscalculia subtypes with similar, low comorbidity profiles: A mixture model analysis (supplementary material)

Christian Kißler, M. A., M. A. [1*], Dr. Christin Schwenk[1], Prof. Dr. Jörg-Tobias Kuhn[1]

[1]Methods of educational research, Faculty of rehabilitation sciences, TU Dortmund, Dortmund, Germany

*** Correspondence:**
Christian Kißler, M. A., M. A.
christian.kissler@tu-dortmund.de

Table 1: Subgroup mean comparison with imputation (ZAREKI-R sample) – clustered by constructs

	Seed = 3			Seed = 3,000			Seed = 3,000,000		
	t-test	Cohen's *d*	BF	*t*-test	Cohen's *d*	BF	*t*-test	Cohen's *d*	BF
Intelligence	1.98	0.42	1.23	2.61*	0.57	4.25	2.35*	0.50	2.45
Reading fluency	1.12	0.24	0.39	1.90	0.41	1.09	2.53*	0.54	3.52
Working memory	1.60	0.34	0.68	1.65	0.36	0.74	1.78	0.38	0.88
Basic numerical processing	5.71***	1.21	$9.83*10^4$	5.32***	1.15	$2.12*10^4$	4.88***	1.04	3865.35
Complex number processing	6.28***	1.34	$1.11*10^6$	6.31***	1.37	$1.24*10^6$	6.28***	1.34	$1.11*10^6$
Calculation	11.37***	2.42	$3.44*10^{16}$	10.87***	2.36	$2.92*10^{15}$	10.92***	2.32	$3.79*10^{15}$
Attention	3.46***	0.74	36.04	3.60***	0.78	54.58	4.02***	0.86	196.09

Note: Interpretation of *p*-values: $*p < 0.05$, $**p < 0.01$, $***p < 0.001$; the significance level was adjusted by the *sequentially rejective Bonferroni test* to prevent the alpha error from accumulating (Holm, 1979); interpretation of BFs (Wagenmakers et al., 2018): BF < 0.33 (moderate evidence for the null hypothesis), BF < 0.10 (strong evidence for the null hypothesis), BF < 0.033 (very strong evidence for the null hypothesis), BF > 3 (moderate evidence for the alternative hypothesis), BF > 10 (strong evidence for the alternative hypothesis), BF > 30 (very strong evidence for the alternative hypothesis).

Table 2: Subgroup mean comparison with imputation (ZAREKI-R sample) – clustered by subtests

	Seed = 3			Seed = 3,000			Seed = 3,000,000		
	t-test	Cohen's *d*	BF	*t*-test	Cohen's *d*	BF	*t*-test	Cohen's *d*	BF
Intelligence	1.61	0.35	0.70	1.61	0.35	0.70	1.61	0.35	0.70
Reading fluency	1.90	0.41	1.08	2.06	0.44	1.41	1.71	0.37	0.80
Matrix span	0.54	0.12	0.25	0.54	0.12	0.25	0.54	0.12	0.25
Verbal span	2.12	0.46	1.58	2.55*	0.55	3.68	2.87*	0.62	7.68
Dot enumeration	4.81***	1.03	3014.75	4.81***	1.03	3014.75	4.81***	1.03	3014.75
Symbolic magnitude comparison	3.40**	0.73	30.69	3.40**	0.73	30.69	3.40**	0.73	30.69
Mixed magnitude comparison	3.21*	0.69	18.38	3.21*	0.69	18.38	3.21*	0.69	18.38
Transcoding	2.68*	0.58	4.92	2.68*	0.58	4.92	2.68*	0.58	4.92
Number sets	3.33*	0.83	118.59	3.33*	0.83	118.59	3.33*	0.83	118.59
Number line	5.78***	1.24	$1.34*10^{5}$	5.78***	1.24	$1.34*10^{5}$	5.78***	1.24	$1.34*10^{5}$
Addition	9.88***	2.12	$2.34*10^{13}$	9.88***	2.12	$2.34*10^{13}$	9.88***	2.12	$2.34*10^{13}$
Subtraction	6.76***	1.28	$2.92*10^{5}$	6.76***	1.28	$2.92*10^{15}$	6.76***	1.28	$2.92*10^{15}$
Multiplication mixed with place holder tasks	6.90***	1.48	$1.72*10^{7}$	6.90***	1.48	$1.72*10^{7}$	6.90***	1.48	$1.72*10^{7}$
Attention	2.74*	0.59	5.72	3.38*	0.73	29.14	3.08*	0.66	12.89

Note: Interpretation of *p*-values: *p < 0.05, **p < 0.01, ***p < 0.001; the significance level was adjusted by the *sequentially rejective Bonferroni test* to prevent the alpha error from accumulating (Holm, 1979); interpretation of BFs (Wagenmakers et al., 2018): BF < 0.33 (moderate evidence for the null hypothesis), BF < 0.10 (strong evidence for the null hypothesis), BF < 0.033 (very strong evidence for the null hypothesis), BF > 3 (moderate evidence for the alternative hypothesis), BF > 10 (strong evidence for the alternative hypothesis), BF > 30 (very strong evidence for the alternative hypothesis).

Table 3: Subgroup mean comparison with imputation (HRT sample) – clustered by constructs

	Seed = 3			Seed = 3,000			Seed = 3,000,000		
	t-test	Cohen's *d*	BF	*t*-test	Cohen's *d*	BF	*t*-test	Cohen's *d*	BF
Intelligence	2.88**	0.69	7.71	3.16**	0.71	9.57	2.30	0.56	2.30
Reading fluency	1.65	0.39	0.78	1.73	0.42	0.88	1.90	0.46	1.14
Working memory	1.65	0.39	0.78	1.77	0.43	0.94	0.94	0.23	0.36
Basic numerical processing	0.23	0.05	0.25	-0.10	0.02	0.25	-0.23	0.05	0.25
Complex number processing	11.59***	2.76	$4.51*10^{14}$	11.95***	2.87	$1.78*10^{15}$	11.20***	2.70	$1.02*10^{14}$
Calculation	8.37***	2.00	$1.41*10^{9}$	8.53***	2.05	$2.60*10^{9}$	8.62***	2.08	$3.74*10^{9}$

Note: Interpretation of *p*-values: $*p < 0.05$, $**p < 0.01$, $***p < 0.001$; the significance level was adjusted by the *sequentially rejective Bonferroni test* to prevent the alpha error from accumulating (Holm, 1979); interpretation of BFs (Wagenmakers et al., 2018): BF < 0.33 (moderate evidence for the null hypothesis), BF < 0.10 (strong evidence for the null hypothesis), BF < 0.033 (very strong evidence for the null hypothesis), BF > 3 (moderate evidence for the alternative hypothesis), BF > 10 (strong evidence for the alternative hypothesis), BF > 30 (very strong evidence for the alternative hypothesis).

Table 4: Subgroup mean comparison with imputation (HRT sample) – clustered by subtests

	Seed = 3			Seed = 3,000			Seed = 3,000,000		
	t-test	Cohen's *d*	BF	*t*-test	Cohen's *d*	BF	*t*-test	Cohen's *d*	BF
Intelligence	1.80	0.40	0.78	2.17	0.48	1.25	2.36	0.54	1.99
Reading fluency	1.33	0.33	0.53	2.29	0.56	2.24	1.52	0.36	0.66
Matrix span	0.15	0.04	0.25	0.45	0.11	0.28	0.40	0.10	0.26
Dot enumeration	0.76	0.18	0.32	2.42	0.60	2.90	1.32	0.32	0.52
Symbolic magnitude comparison	0.46	0.11	0.27	1.61	0.40	0.75	0.40	0.10	0.26
Mixed magnitude comparison	-0.09	0.02	0.25	0.10	0.02	0.25	0.99	0.24	0.37
Transcoding	10.94***	2.67	$3.62**10^{13}$	10.72***	2.64	$1.55*10^{13}$	9.83***	2.36	$4.79*10^{11}$
Number sets	3.76**	0.92	75.04	3.72**	0.92	67.04	4.56***	1.10	864.86
Number line	7.90***	1.71	$5.94*10^{6}$	7.42***	1.57	$5.71*10^{5}$	8.35***	1.85	$1.01*10^{8}$
Addition	5.84***	1.43	$7.37*10^{4}$	5.68***	1.40	$4.01*10^{4}$	5.56***	1.34	$2.68*10^{4}$
Subtraction	5.87***	1.44	$8.29*10^{4}$	5.90***	1.45	$9.26*10^{4}$	5.36***	1.29	$1.31*10^{4}$
Multiplication mixed with place holder tasks	5.34***	1.31	$1.21*10^{4}$	6.32***	1.56	$4.45*10^{5}$	6.14***	1.47	$2.23*10^{5}$

Note: Interpretation of *p*-values: $*p < 0.05$, $**p < 0.01$, $***p < 0.001$; the significance level was adjusted by the *sequentially rejective Bonferroni test* to prevent the alpha error from accumulating (Holm, 1979); interpretation of BFs (Wagenmakers et al., 2018): BF < 0.33 (moderate evidence for the null hypothesis), BF < 0.10 (strong evidence for the null hypothesis), BF < 0.033 (very strong evidence for the null hypothesis), BF > 3 (moderate evidence for the alternative hypothesis), BF > 10 (strong evidence for the alternative hypothesis); BF > 30 (very strong evidence for the alternative hypothesis).
